# Milled versus moulded mock-ups based on the superimposition of 3D meshes from digital oral impressions: a comparative in vitro study in the aesthetic area

**DOI:** 10.1186/s12903-019-0922-2

**Published:** 2019-10-29

**Authors:** Francesca Cattoni, Giulia Teté, Alessandro Mauro Calloni, Fabio Manazza, Giorgio Gastaldi, Paolo Capparè

**Affiliations:** 1 Dental School, Vita-Salute San Raffaele University, Milan, Italy; 2 Department of Dentistry, IRCCS San Raffaele Hospital, Milan, Italy; 3 Specialisation School in Oral Surgery, Vita Salute San Raffaele University, Milan, Italy; 4 Unit of Oral Maxillofacial Surgery, San Rocco Clinical Institute, Ome, Brescia, Italy

**Keywords:** Digital planning, Digital smile design, Mock up, Milling mock up, Digital workflow

## Abstract

**Background:**

Aesthetic porcelain veneers proved to be a long-term reliable prosthetic solution, ensuring minimal invasiveness. The use of veneers requires an adhesive cementation technique, so maintaining as much enamel as possible is to ensure lasting success. A diagnostic mock-up is a key tool that allows a preview of the outcome of the aesthetic restoration: it is obtainable both in an analog and digital way. With the recent developments in impression technology and the ever so fast growing use of CAD-CAM technologies it is useful to understand the pros and cons of either one of these techniques (analog and digital) in order to identify the easier and more convenient workflow in aesthetic dentistry.

**Methods:**

After taking pictures and impressions of the dental arcs of a patient in need of aesthetic rehabilitation, 52 resin models were produced and a digital drawing of the smile was outlined. Both an analog and a digital wax-up were obtained from two of the 52 models: the latter was obtained using digital impressions and a dedicated software. The analog wax-up was then used to produce 25 matrices that have later been used to mould 25 resin mock-ups using a traditional moulding protocol (Control Group - CG). The digital wax-up was used to mill 25 PMMA mock-ups. Each mock-up, both milled and moulded (total 50), was then laid on the other 50 resin models as a digital impression of it was taken. The STL. files of the milled mock-ups were compared with the 3D CAD wax-up made using a specific software. The STL. files of the analog printed mock-ups were compared with the traditional wax-up design. A statistical analysis was carried out to evaluate the difference between the groups.

**Results:**

The statistical analysis showed a significant difference (*P* > 0.01) between the mean value of the distance between the points of the overlapping STL. meshes in GC (0.0468 mm) and in TG (Test Group - TG) (0,0109 mm).

**Conclusions:**

The study showed a difference in accuracy between traditional moulded and milled mock-ups compared to their original wax-up. The data analysis reports that the digital method allows for greater accuracy. Within the limitations of this study, a fully digital workflow is to considered more reliable when it come to creating an esthetic mockup: the digital procedure has been shown to be more accurate than the one made manually which is much more operator dependent and it brings an increase to the chance of error, and that could ultimately affect the final result.

## Background

In recent years, the expectations of dental patients regarding aesthetic appearance have increased greatly. Aesthetic results have already reached comparable importance to masticatory function [1, 2]. Porcelain veneers proved to be a long-term reliable solution, ensuring maximum aesthetics success and minimal invasiveness [3, 4]. It is a priority to the clinician to pursue the least invasive procedure in every prosthetic restoration, preserving as much natural tooth structure as possible and respecting surrounding soft tissues [5]. Furthermore, since the use of veneers requires an adhesive cementation technique, maintaining as much enamel as possible is to ensure lasting success [6]. A diagnostic mock-up is to be intended as a tool that allows a better understanding of the patient’s aesthetic expectations previewing the outcome of the aesthetic restoration, at a stage where it is still very easy to make changes according to patient’s requests. It improves the communication with the patient, allowing prosthetic restorations to be achieved more successfully ( [7, 8]). Moreover, a protocol that uses a diagnostic mock-up to guide the preparation has proved to be more conservative than a classical non-guided preparation made by the clinician [9]. As reported by Magne et Al., a veneer preparation driven by the final volume of the restoration (a diagnostic mock-up) allows for more enamel preservation, avoiding unnecessary over-preparation by only removing the structure needed to create proper prosthetic thicknesses, and more predictable outcome in terms of bonding, biomechanics and final aesthetics [10]. According to Coachman’s protocol, the realization of the diagnostic wax-up is preceded and guided by the Digital Smile Design, which has proved to be a fundamental and useful tool for improving communication and patient’s acceptance of the dental procedure [11–15]. This articulated workflow requires several steps that can lead to various inaccuracies. For instance, the mock-up molding phase on the existing tooth appears to be a very complex and heavily operator-dependent process. The most common problems related to the resin mock-ups are: the unevenly balanced positioning of the matrix, the inhomogeneous pressure during resin hardening, the difficulty in remove excess resin and the while finishing part to get a good final result [10]. Result that, if excessively discordant from what was promised and evaluated with the patient through the DSS software preview, could cause communication problems, misunderstanding and disappointment, or even the need to repeat the procedure, causing a waste of time and increasing the number of appointments required. Today, in attempt to minimize the chance of error and to shorten working times, the clinician can rely on effective smile planning tools and CAD / CAM systems (3D-Lynx Srl. Varese, Italy). Such systems, as shown by McLaren et Al., have proved their reliability in the realization of adhesive restorations in aesthetic areas [16]. A dedicated digital smile planning software with both two-dimensional and three dimensional features, is able to obtain excellent results in a simple, standardized and less operator-dependent way (3D-Lynx Srl. Varese, Italy). The aim of this study is to evaluate the traditional mock-up production method, which involves mock-up molding with a silicone matrix of the wax-up, compared to an exclusively digital workflow, which consists of mock-up milling from a CAD design, based on a digital optic impression. The accuracy of the two different types of mock-ups was compared each to their specific design and diagnostic wax-up.

## Methods

**Fig. 1:**
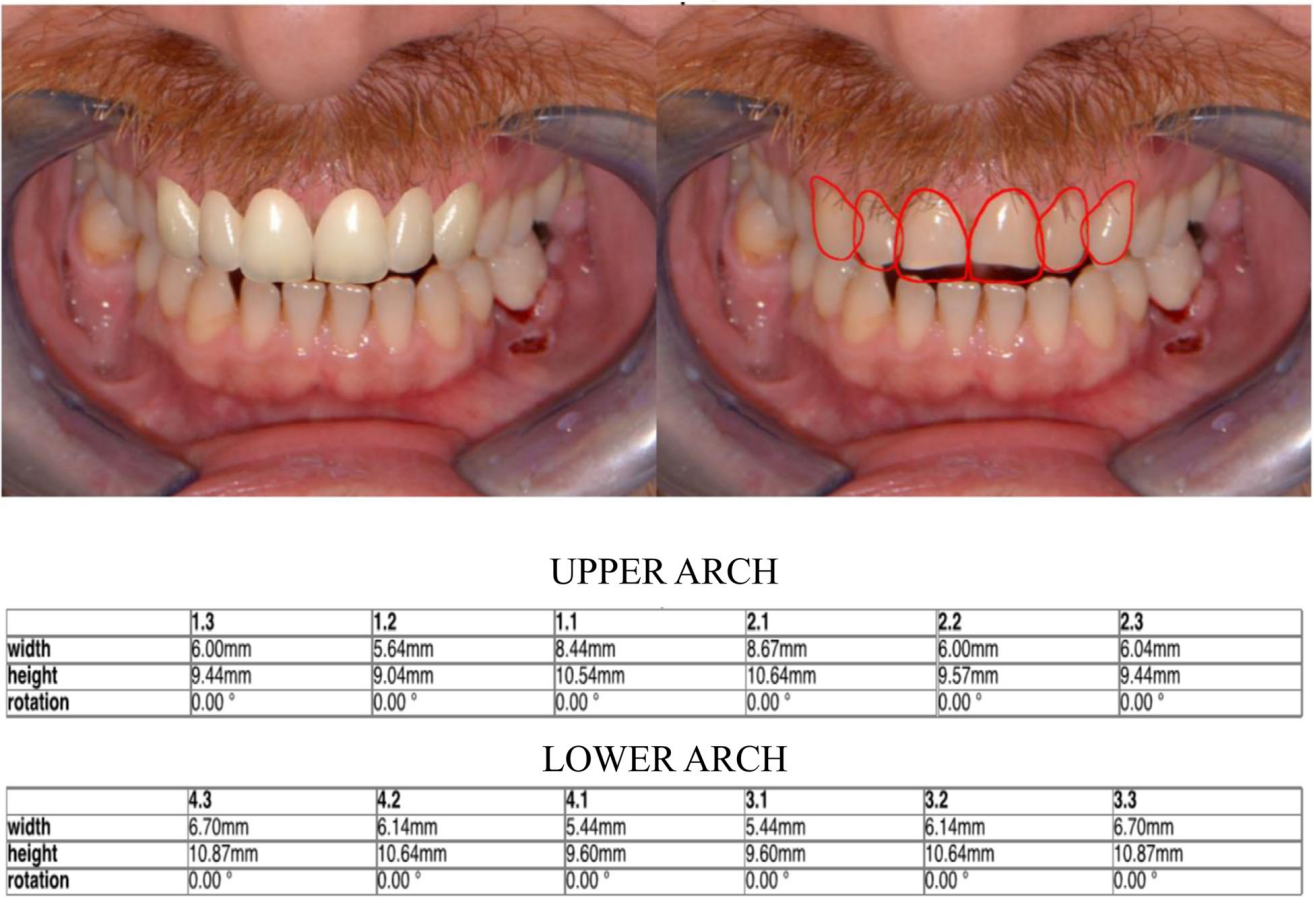
The DSS report. All the numeric measurements of the digital design are recorded and can be sent to the technician for a more efficient communication

**Fig. 2:**
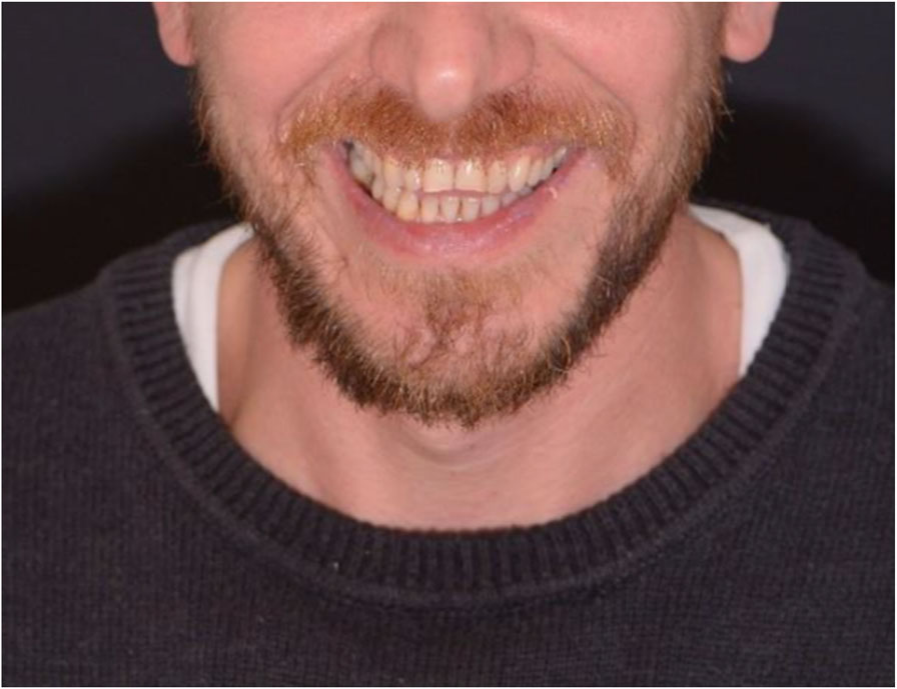
Extra-oral photograph with with maximum smile

**Fig. 3:**
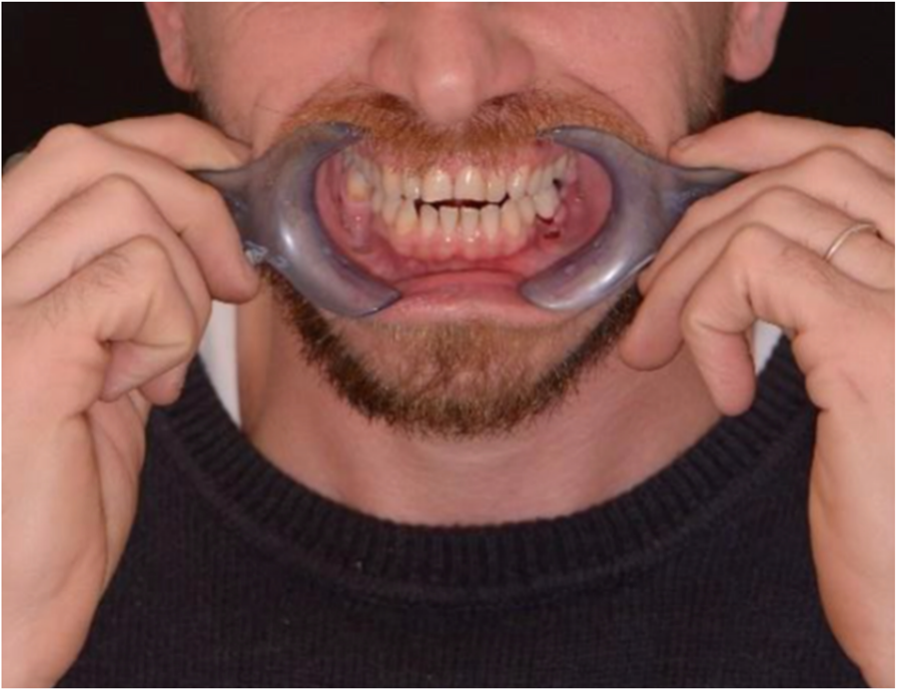
Intraoral photograph with slightly spaced teeth, with specific glasses

**Fig. 4:**
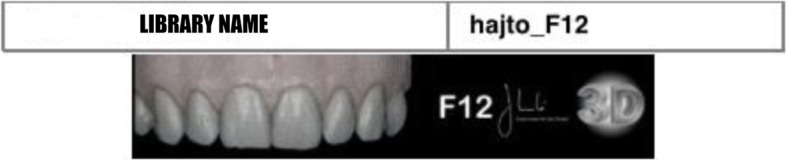
The selected 3D teeth library

**Fig. 5:**
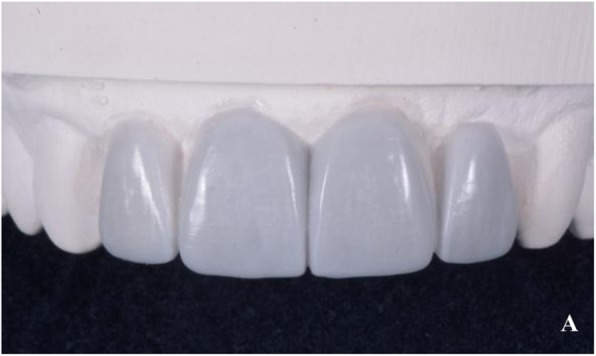
Diagnostic mock-up

A patient (male) in need of an additive restoration in the anterior area was selected in the dentistry department of IRCCS San Raffaele Hospital. Diagnostic pictures where taken during the first appointment, as well as analog polyether impressions of the upper arch (Impregum Penta, 3 M ESPE, Sain Paul, Minn. USA). The virtual drawing of the final restoration was then obtained using a digital smile design software called DDS-2D (3D-Lynx Srl. Varese, Italy) (Fig 1). Starting from two photos of the patient, a extraoral shot (Fig. 2) with a maximum smile and an intra-oral one with slightly disclosed dental arches (Fig. 3) a digital drawing of the new smile was obtained and shown to the patient. This drawing was based on standard dental shapes included in a library inside the 2D software (Fig. 4). The digital restoration project was then realized (Fig. 5). This study was approved by the ethical board of the IRCCS San Raffaele Hospital of Milan (9/INT/2015). The patient provided their informed consent in writing.

### Traditional wax-up (control group)

**Fig. 6:**
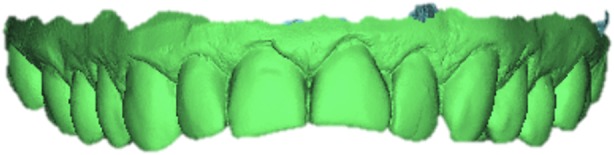
STL file of the initial situation

**Fig. 7:**
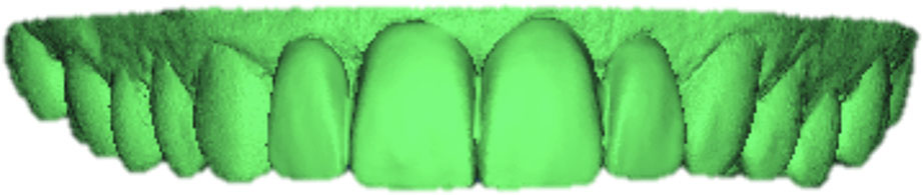
CAD design of the digital wax-up

Fifty-two resin models were obtained from the analog impressions: one of them was used to produce a traditional wax-up by the technician, one was scanned using a laboratory desktop scanner (Scanner S-6000, Zirkonhzan Srl, Gais BZ) finally obtaining a STL file (Fig. 6) that allowed the design of a diagnostic mock-up using the Smile Design Software 3D-CAD expansion DSS-3D (3D-Lynx s.r.l., Varese, Italy). For the analog diagnostic wax-up, the software report provided the technician with all the images (Fig. 1) and all the operations performed by the clinician, indicating, with linear measures, how lengthened or shortened each measure had been during the design process. A intra-oral scanner (Dental Wings Intraoral Scanner, Dental Wings, Montreal, Canada) was then used to generate a STL file of the wax-up itself (Fig. 7).

**Fig. 8:**
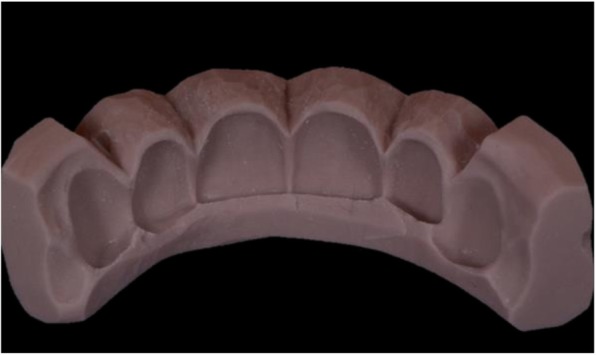
The silicon index used to mould the mock-up

**Fig. 9:**
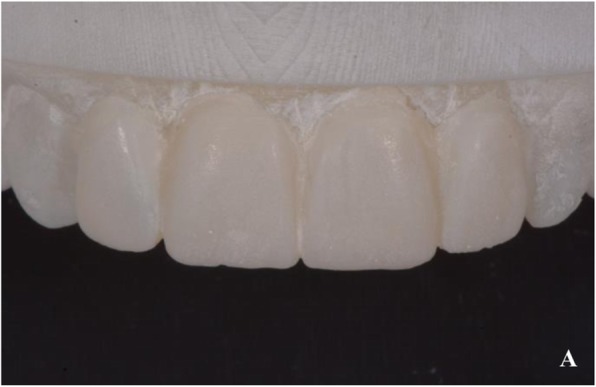
One of the moulded mock-ups

Twenty-five silicone matrices (Fig. 8) were produced starting from the traditional wax-up (Fig. 9) (CG - Control group) and they were then used to mould a dual curing compost resin mock-up (Protempt 4, 3 M ESPE, Saint Paul, Minn. USA) on 25 of the remaining 50 resin models. The other 25 were used as a base for the 25 milled resin mock-ups to be laid upon (TG - Test Group). Those milled resin mock-ups were obtained by the STL - CAD project. Finally a digital impression of each of the 50 mock-ups (both moulded and milled) was taken with an integral scanner (Dental Wings Intraoral Scanner, Dental Wings, Montreal, Canada) and later analyzed.

### Digital wax-up (test group)

**Fig. 10:**
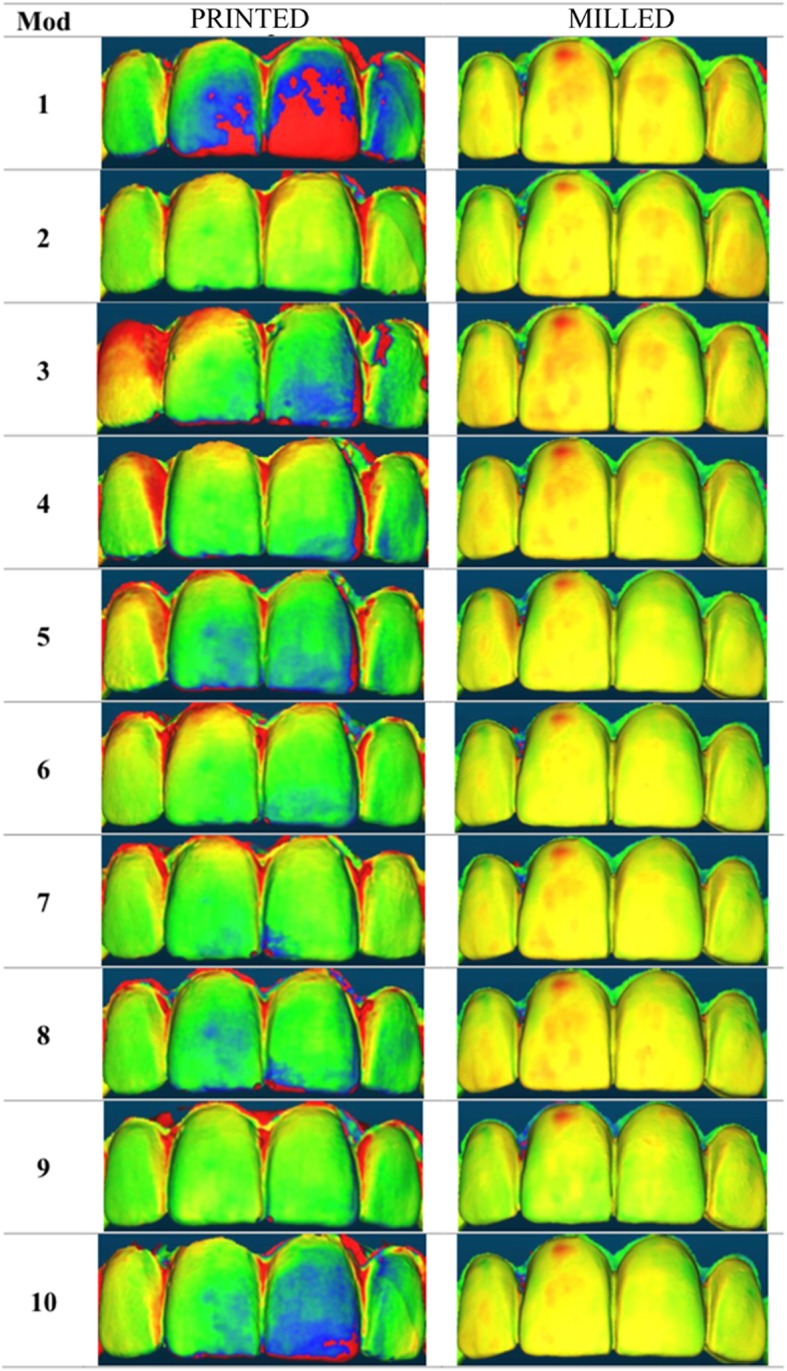
Graphical evaluation of printed and milled mock-up overlays for each of the 10 respective models in occlusal view, made with CloudCompare software

In order to obtain a digital wax-up, the STL. files of the patient’s dental arches were uploaded to DSS 3D, a software which is the direct 3D implementation of the aforementioned DSS 2D. This software is able to align the patient’s photograph to the digital model and support. The CAD software allowed us to design a three-dimensional digital wax-up directly on the model. 25 resin mock-ups (PMMA - polymethil methacrylate) were then milled from the from the digitally planned 3D wax-up. Each of these were then laid on the other 25 plaster models and as an optical digital impression was detected with the aforementioned intra-oral scanner. All the digital impressions of milled mock-ups, the moulded mock-ups, the wax-up and the STL. file of the CAD design were then uploaded to a lab software (OpenText Exceed 2017-EIM-Waterloo, ON,Canada). A superimposition and segmentation of the digital files have been performed to ensure that the impressions were cut out all the same way so the comparisons could not be affected by the different extensions of the original scans. Using a comparison software by (CloudCompare, https://www.cloudcompare.org) (Fig. 10), the STL files of the digital impressions, of the 25 manually moulded mock-ups were compared with the digital impressions of the traditional analog diagnostic wax-up while the STL files of the milled mock-ups were compared with the CAD designed project made with CAD - DSS 3D software (3D Lynx - Varese, Italy). The Stl. Files were segmented to make sure that only the area of the wax up was taken into consideration during the comparison. This was done by overlapping the two meshes with a “three point” manual alignment technique and measuring the average distance between the points of each one. This procedure represented also an index of the actual volumetric difference between them. Finally, the two methods were compared, evaluating which carried more errors and which remained more faithful to their design. Statistical analysis was carried out with SPSS-Student T-test, which allows to compare the mean values of two non-coupled data sets.

## Results

Results has shown specific areas of accumulation of errors and deviations: the cervical margin and the incisal edge (Fig. 10). The reasons why these particular areas exhibited major alterations are likely to be that the area of ​​the cervical margin is the point where the excess of resin is ​​removed from the silicone matrix, a procedure that is understandably difficult to replicate, while the differences found at the incisal edge are plausibly associated with variations in the pressure exerted by the operator on the silicone matrix to keep it in place during the hardening phase of the resin. The incisal edge, in particular, is of great aesthetic importance since it is a focal point for the observer; excessive variations in this area between the design and mock-ups can surely upset the patient, who is able to perceive the diversity of what has been promised with what he is really trying in his mouth.

**Fig. 11:**
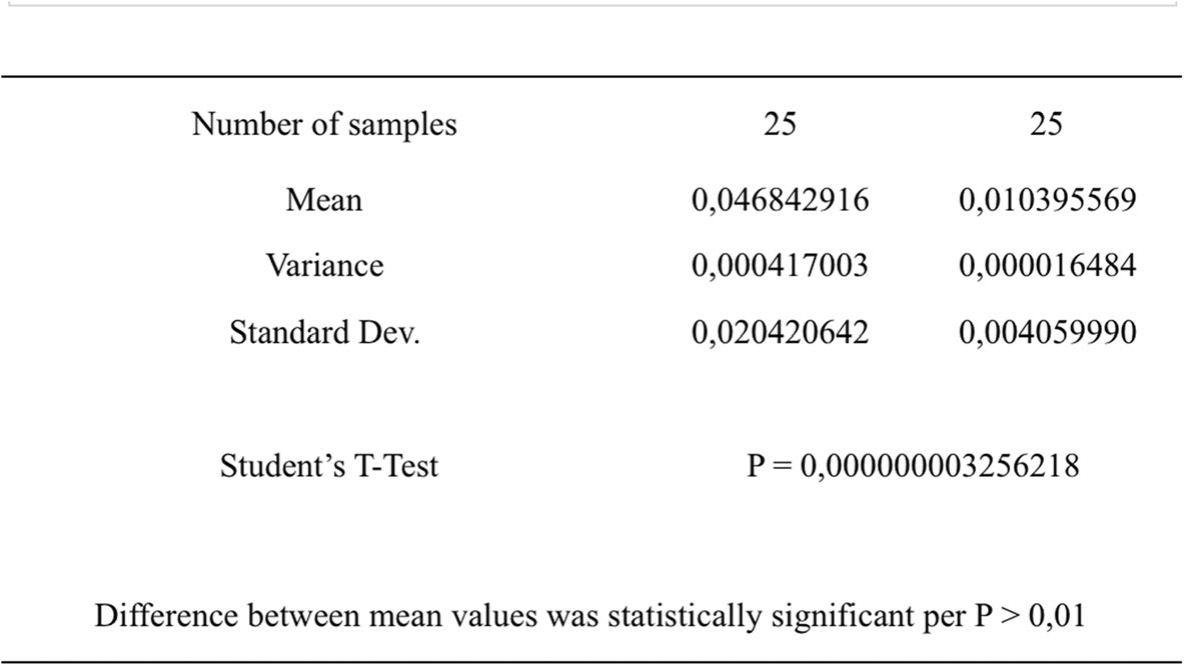
Comparing mean values of two non-coupled data sets, with different variance

The statistical analysis showed a significant difference between the two types of mock-ups. The null hypothesis that claims that the differences between the two groups were due to chance must be therefore rejected. The result obtained thus showed a clear difference in accuracy between moulded and milled mock-ups compared to their design. In the first case, not only moulded mock-ups diverged significantly from the diagnostic wax-up, demonstrating less accuracy (how much a measure is close to the true value of the size), but they also denoted a lesser degree of precision (how much measures are close to each other), indicated by the variance compared to the sample average (variance of moulded mock-ups: 0.0004). The milled mock-ups, on the contrary, were much more faithful to their CAD design, since the only error existing is that of the milling machine during the production phase, certainly negligible (variance of milled mock-ups: 0.00002) (Fig. 11). The milled mock-ups were therefore more accurate and precise. The use of the software allowed the comparison between the mean value of the distance between the points of the meshes superimposed on each other. Comparing the results obtained from the 50 evaluations performed, it was graphically very clear how the degree of overlap between moulded mock-ups and the diagnostic wax-up was significantly lower than that between milled mock-ups and the relative CAD design (Fig. 10).

From a more careful assessment, it was noted that the areas of accumulation of major deviations, which reflect errors during the realization, were consistently related to the portion of the cervical margin and the incisal edge.

**Fig. 12:**
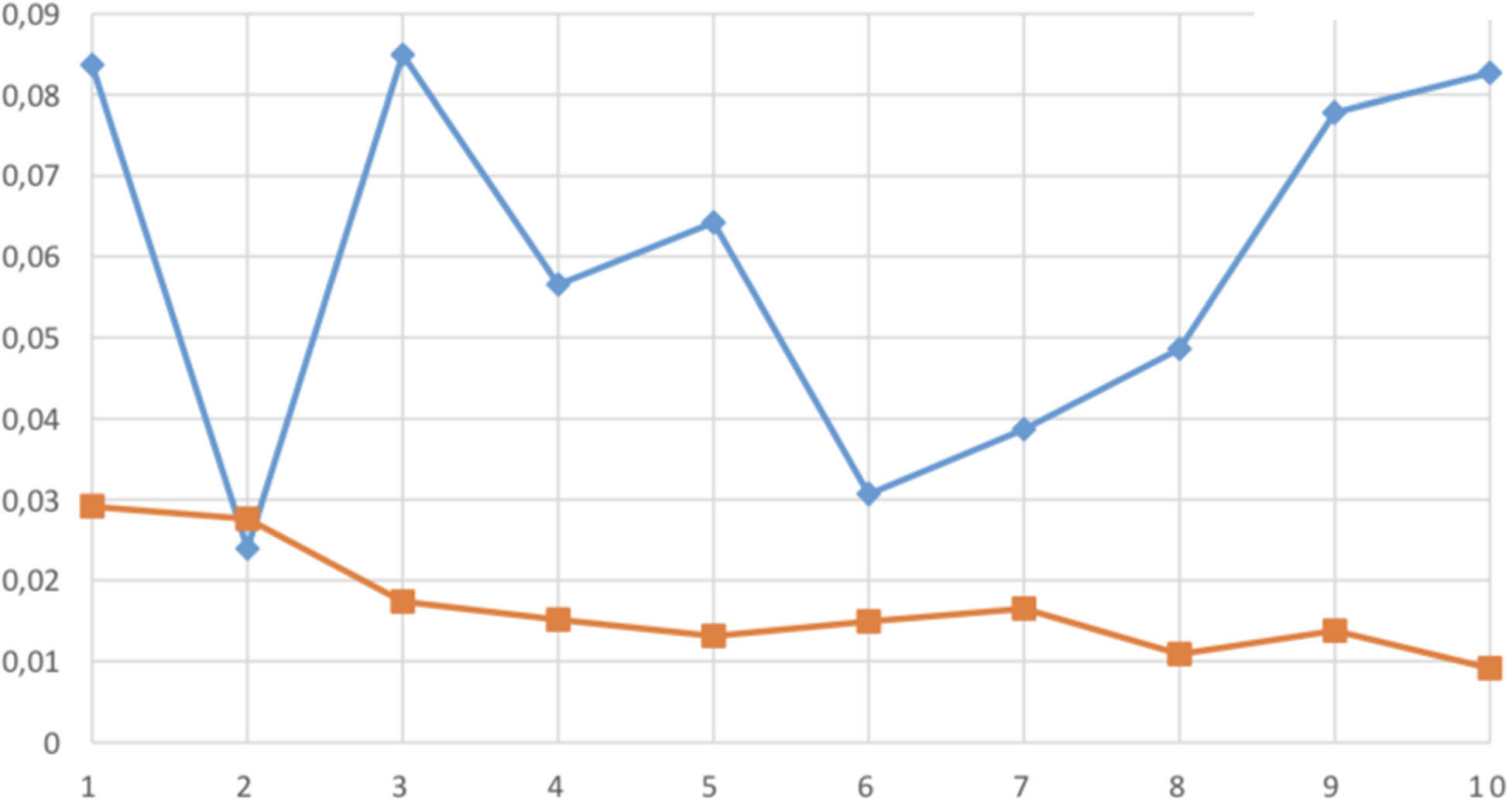
Linear distribution of medium distances between points of the two meshes expressed in millimeters (blu line: average distance in the printed group, orange line average distance in the milled group). The graph points out a higher variability. In the moulded group and a higher deviation of the measurements compared to the milled group. This means that moulded mock ups tend to vary in shape and measurements from the original design of the wax up a lot more than the milled ones

The statistical analysis showed that the difference between mean value of the distance between the points of the meshes superimposed in the moulded mock-up group (0.0468 mm) compared to the milled mock-up group (0.0109 mm) was statistically significant (*P* < 0.01; *P* = 0.300000000326) (Fig. 12).

## Discussion

The aim of this study is to evaluate which method allows for the closest match with the initial wax up design and to find which stage of the production is affected by the greatest loss of precision, compromising the final result. In addition to the geometric and volumetric comparison between the different mock-ups and the respective wax-ups, the ease of execution for an unexperienced user has also been evaluated for each technique, since all the tests were carried out by an inexperienced operator. Many studies have shown how a mock up based approach can enable the clinician to provide patients with predictable aesthetics, since this particular kind of tool works with the psychology of patients, improving their attitude and compliance towards the treatment [10]. When starting a case, it is best for the practitioner to have in mind the end result since this has been shown to be vital in cases where the anterior teeth morphology is to be changed. A diagnostic wax-up can enhance the predictability of treatment by modeling the desired result in wax prior to treatment. It is critical to correlate the wax-up to the patient to avoid a result that appears optimal on the casts but does not correspond to the patient’s smile [15–24]. Sancho-Puchades et al., point out that the use of a mock up will only be effective in an additive reconstructive case, while in subtractive cases it has to be used later in the treatment, after a minimum preparations of the natural teeth [25]. Wax ups and mock ups are reported to be extremely useful also for periodontal surgeons as tools used to perform crown lengthening procedures to enable future restorations in specific cases [13]. Results has shown specific areas of accumulation of errors and deviations; the cervical margin and the incisal edge. The reasons why these particular areas exhibited major alterations are likely to be that the area of the cervical margin is the point where the excess of resin is removed from the silicone matrix, a procedure that is understandably difficult to replicate, while the differences found at the incisal edge are plausibly associated with variations in the pressure exerted by the operator on the silicone matrix to keep it in place during the hardening phase of the resin. From the perspective of the impression techniques used in this study, the results showed that a digital workflow was to be considered preferable in the hands of an unexperienced operator. Studies in literature claim that even though material such as poly-vinyl siloxane present great accuracy, digital impression techniques seem to be superior in terms of time and material saving; at the same time, said techniques lack in repeatability and this aspect represents a problem in need of solution [2]. As it was shown by the works of Gherlone et al. digital impression techniques manage to create an accurate physical model significantly improving efficiencies for the dental team and streamlining the workflow [17]. As far as the whole digital technique is concerned, a big role is played by the preview of the final result obtained through Digital Smile Design protocol. The use of a smile designing software allows for an interdisciplinary collaboration between practitioners and this seems to improve the decision making process, ultimately decreasing the amount of intra-oral adjustments [12–28]. This tool allows the patient to preview the prosthetic result directly on a picture; it also provides the dental technician with all the necessary information on the execution of the work through a detailed report.

## Conclusions

The study showed a difference in accuracy between traditional moulded and milled mock-ups compared to their original wax-up. The data analysis reports that the digital method allows for greater accuracy. Compared to the milled ones, the use of moulded mock ups would resolve in less accuracy of the mockup itself making it more difficult for the patient to visualize the final result while wearing it therefore compromising the validity and acceptance of the entire prosthetics treatment plan. Within the limitations of this study, a fully digital workflow consisting in digital impression, digital wax up and milling technology is to considered more reliable when it comes to creating an esthetic mockup: the manual procedure has ben proven to be much more operator dependent and it brings an increase to the chance of error, and that could ultimately affect the final result.

## Data Availability

All materials described in this manuscript including all relevant raw data, will be freely available to any scientist wishing to use them for non-commercial purposes, without breaching participant confidentiality. The data of this research is available from Paolo Capparè (corresponding author).

## Abbreviations

CG: Control Group
PMMA: Polymethil methacrylate
TG: Test Group
